# Editorial: Women's Lung

**DOI:** 10.3389/fmed.2021.704980

**Published:** 2021-06-30

**Authors:** Joanna Domagala-Kulawik, Dragana Jovanovic, Chantal Raherison-Semjen

**Affiliations:** ^1^Department of Internal Medicine, Pulmonary Diseases and Allergy, Medical University of Warsaw, Warsaw, Poland; ^2^Internal Medicine Clinic “Akta Medica”, Belgrade, Serbia; ^3^Epicene Team U1219, INSERM, University of Bordeaux, Bordeaux, France

**Keywords:** lung, women, lung cancer, estrogens, smoking

The high incidence of chronic lung diseases generates a substantial health problem worldwide (ERS White Book, https://www.erswhitebook.org/). Recently, increasing recognition of the importance of the disease's phenotypes was noted. Most lung diseases are regarded as a whole. In the epidemiological reports, the attention is focused on age and race rather than sex, although there is evidence that sex influences the expression of respiratory disease as well. The epidemiology of lung diseases, the susceptibility to risk factors, and the clinical course of the disease all differ in males and females. The aim of separate consideration of lung diseases in the female population is possibly a subsequent modification of diagnostic procedures and establishing additional specific criteria in the treatment approach, which might result in specific prophylactic programs and targeted therapies. It would be in line with modern personalized and precision medicine. Recent recognition of the specific and different course of lung cancer and chronic obstructive lung disease (COPD) in the female population represents a good example (1, 2).

With evidence that female phenotypes are often under-recognized and under-treated, addressing the “female lung” is an important challenge. On the basis of this first-of-its-kind Research Topic, we would like to better the collective understanding of the value of the recognition of the role the female sex has in chronic lung diseases and to translate this knowledge into clinical practice. This will require time and complex research studies. Some respiratory scientific societies already have a section dedicated to diseases in females, France being a prime example (https://splf.fr/femmes-et-poumon).

The use of proper medical terminology in the exchange of knowledge is crucial. This applies, for example, to the terms of sex and gender, which are not the same. “Sex” refers to biology and “gender” to behavioral and social determinants (3, 4). On the one hand, sex is an influencing factor on gender; on the other hand, gender influences the modification of epigenetic changes and thus the risk of some diseases. The use of terminology requires caution, and this should be kept in mind during study planning.

Most of the articles submitted to Research Topic Women's Lung are reviews, showing that the authors are familiar with this problem, but original research studies are, in many aspects, urgently needed. Most articles in this Issue describe risk factors for female lung diseases, such as smoking, genetics, hormonal status, and the microbiome.

Interestingly, the subject of one article is currently very important, and it relates to smoking habits and COVID-19. Milhatan et al. have conducted a systemic review on the impact of tobacco smoking on SARS-CoV-2 infection and COVID-19 disease. Smoking has been shown to be a risk factor for COVID-19 by increasing susceptibility to infection and modulating the expression of angiotensin-converting enzyme 2 (ACE-2). The authors present their results compared with the existing literature, meta-analyses on the relationship between smoking and COVID-19 incidence, hospitalization, disease severity, mortality, and prevention in the context of biological sex. With one exception, cigarette smoking has been found to increase the severity of the COVID-19 disease and worsen prognosis in males. The higher proportion of smokers among males is one of the possible explanations, and others could involve better hygiene and the protective role of estrogens in females. Authors have concluded that in the time of the SARS-CoV-2 pandemic, an additional indication for antinicotine programs exists, especially among women. This shows a new direction for future studies.

Estrogens and lung cancer is a subject of review by experienced authors in the field, such as Rodriguez-Lara and Avila-Costa Sexual dimorphism causes different clinical characteristics of lung cancer in females vs. males, different distributions of histological types, and different genetic profile. The authors present different risk factors for female lung cancer apart from well-known active smoking, which is still popular among young women, including the following: second-hand (passive) smoking, wood smoke exposure, inhalation of cooking-derived products, and ambient air pollutants. The role of estrogens, estrogens receptors, and the enzymatic pathway of estrogen production by aromatase in the lung and carcinogenesis was described in detail and illustrated comprehensively. In spite of the role of estrogens in carcinogenesis, there is no direct impact on relation of hormonal status and lung cancer course and treatment outcomes. However, aromatase inhibitors and inhibitors of estrogens receptors were effective in experimental studies and clinical trials.

The influence of sex on chronic lung diseases by modification of the gut–lung microbiome was a topic of a comprehensive study by Beauruelle et al. The authors use the term “microgenderome,” showing, on the basis of many observations and animal models, that the airways (in connection with the gut) are characterized by the steady-state microbiome. The gut–lung axis is capable of modifying the immune system. Thanks to new genetic techniques, the knowledge on lung microflora has developed, showing that the lung is not sterile. It was shown by the authors that the distribution of lung diseases differs between the male and female populations, with the prevalence of chronic entities being shown among females. There is a different distribution of pathogens in cystic fibrosis: the most serious are observed in the female sex, but the acute community-acquired infections are most often noted among males. The role of estrogens in the modification of anti-inflammatory host defense is well-established: these hormones enhance the immune response, change mucus properties and thus providing a more protective function, and modify the bacterial–host interaction. To summarize, this article represents a basis for further studies that are very important for possible modification of treatment and prevention of chronic respiratory diseases.

Endometriosis belongs strictly to female diseases of the respiratory tract. Wang et al. described in their article a case report confirmed by data from literature concerning endometriosis-related pleural effusion. They highlighted that this clinical problem should be taken into account by physicians in the case of spontaneous pneumothorax. Interestingly the authors present confirmation of endometriosis-related pleural effusion by cytological examination. The endometrial origin of the clusters of glandular cells could be confirmed by a positive reaction to estrogen and progesterone receptors. The results of the literature search showed that endometriosis-related PE is observed in young women aged 35+/−8 years old, the main symptoms being catamenial chest pain, dyspnea, and cough (50.7,74.6, and 26.9% respectively). The chest CT findings include pleural effusion (79.1% unilateral), pleural thickening (8.6%), and often pneumothorax.

Sexual dimorphism of the respiratory tract begins *in utero*. Tremblay and Morin-Labbé present their experimental model, which illustrates the local production and action of androgens in lung development. This is a model of intracrinology, e.g., “production of active sex steroids *in situ* within the cells where the action takes place”. In these experimental models, androgen receptors were found with similar distribution in males and females, and the local activity of steroids and sex hormones in lung development as well as causes of bronchopulmonary dysplasia are presented. These results correspond well to the opinion that inappropriate lung development might be responsible for obstructive lung diseases in adults^1^.

To conclude, the idea of a Research Topic dedicated to lung diseases in females turned out to be interesting for authors and revealed a wide range of issues worth considering for further research and popularization.

## Author Contributions

All authors listed have made a substantial, direct and intellectual contribution to the work, and approved it for publication.

## Conflict of Interest

The authors declare that the research was conducted in the absence of any commercial or financial relationships that could be construed as a potential conflict of interest.

## Footnotes

^1^http://www.goldcopd.org.

## References

[1] Domagala-Kulawik J Trojnar A. Lung cancer in women in 21th century. J Thorac Dis. (2020) 12:4398–410. 10.21037/jtd-20-287 32944353 PMC7475544

[2] Gut-Gobert C Cavaillès A Dixmier A Guillot S Jouneau S Leroyer C. Women and COPD: do we need more evidence? Eur Respir Rev. (2019) 28:180055. 10.1183/16000617.0055-2018 30814138 PMC9488562

[3] Bewley S McCartney M Meads C Rogers A. Sex, gender, and medical data. BMJ. (2021) 372:n735. 10.1136/bmj.n735 Erratum in: *BMJ*. (2021). 33741563

[4] Mauvais-Jarvis F Bairey Merz N Barnes PJ Brinton RD Carrero JJ DeMeo DL. Sex and gender: modifiers of health, disease, and medicine. Lancet. (2020) 396:565–582. 10.1016/S0140-6736(20)31561-0 Erratum in: *Lancet*. (2020) 396:668. 32828189 PMC7440877

